# Food-Based Dietary Guidelines and Protein Quality Definitions—Time to Move Forward and Encompass Mycoprotein?

**DOI:** 10.3390/foods11050647

**Published:** 2022-02-23

**Authors:** Emma Derbyshire

**Affiliations:** Nutritional Insight, Surrey KT17 2AA, UK; emma@nutritional-insight.co.uk

**Keywords:** food-based dietary guidelines, global mycoprotein, planetary health, protein, protein demands, protein diversity, protein quality

## Abstract

Food-Based Dietary Guidelines (FBDG) lack uniformity globally, with the integration of protein food sources being highly variable. Protein guidance tends to be dichotomous, e.g., animal versus plant with other categories such as fungal proteins being overlooked. In 2019 the EAT Lancet Food in the Anthropocene report was a chief driver questioning the need to supply healthy diets from sustainable food systems. Some countries are developing FBDG that integrate these aspects, but these are quite often protracted, too subtle or misaligned with other countries, diluting the effects of meaningful global change. Protein quality metrics also underpin the dissemination of dietary guidance. However, for protein, these remain based on a food’s essential amino acid profile and digestibility scores, thus are nutritionally and physiologically centric. It has been proposed that this definition is becoming increasingly myopic from a wider societal perspective. Updated indices should include contemporary issues such as protein diversity and environmental outcomes. Taken together, there is opportunity for renewed thinking about both FBDG and protein quality definitions, with scope to include both health and environmental outcomes and need to move towards the concept of protein diversification.

## 1. Introduction

We are now in the era of anthropogenic climate change, with the Conference of the Parties (COP) 26 and prior meetings recognising that developments in agriculture will be crucial to moving forward and meeting targets to cut emissions [1]. As advised by the Intergovernmental Panel on Climate Change, to keep global warming < 1.5 °C we must urgently reflect on what we eat and how these foods impact on the environment [2]. Subsequently, times are changing when it comes to the forms of food proteins that we consume, with plant and fungal proteins gaining particular attention [3,4]. In the 21st century, trends towards flexitarianism, vegetarianism and veganism are accelerating in popularity, particularly in the United Kingdom (UK) [5]. Data from the UK National Diet and Nutrition Survey (NDNS) shows that since this online survey first commenced (in 2008) there has been a decline in mean red and processed meat intakes across all age groups [6,7]. This downward trend was reinforced by Stewart et al. [8], who observed that an acceleration of this trend is still needed to achieve meat-consumption targets aligned with sustainable diets.

From a global perspective, meat consumption is also changing. An analysis of data from 35 countries between 2009 and 2019 showed that certain countries—New Zealand, Canada and Switzerland—are reaching a plateau of peak meat consumption [9]. In general, over these 35 countries, poultry consumption increased whilst that of beef and lamb/mutton declined [9].

There are an array of factors propelling the need to change our dietary protein profiles. Firstly, increased life expectancies coupled with expanding global populations are driving greater demands for dietary protein [10,11]. For example, it has been projected that by 2050 the global demand for animal products will rise by 60–70% (predominantly fuelled by low- and middle-income countries) [12]. Projected trends in animal-sourced foods confirm that major changes in animal production and protein supply are required to ensure future sustainability of protein intake [13]. Secondly, growing concerns about the impact of what we eat on human and planetary health boundaries is driving calls to curtail the consumption of ‘animal-derived’ proteins [10]. Livestock, for example, not only adversely affects greenhouse gas (GHG) emissions but also water footprints and pollution [14]. In 2019 the EAT-Lancet Food in the Anthropocene report was a milestone publication, emphasising the urgent need to feed growing global populations with diets that are both healthy and sustainable [15]. This publication was an important driving force accentuating the urgent need for change. The mentioned underpinned combination of factors create a “perfect storm” and necessitate the need for sustainable food protein sources [16].

It has been proposed that in the foreseeable future, the protein economy will need to be ‘mixed’ and contain a spectrum of food proteins [10]. To safeguard protein sustainability for a growing global population, there is increasing interest in the role of fungi as a protein source [4,10]. The renowned epidemiologist Professor Walter Willett and coauthors (2021) report that there is now a “pressing” need to formulate dietary guidelines for healthy and sustainable diets [17]. Yet, sadly, this is not advancing with the rapidity needed. Dated protein quality definitions and inconsistent global Food-Based Dietary Guidelines (FBDG) mean that many global public health messages are outdated, fragmented and lacking in consensus. The current publication discusses where we are at presently with protein quality definitions, FBDG, and describes where we need to be in the immediate future.

## 2. Fungal Proteins and Mycoprotein

Interest in fungal-derived mycoproteins has been gaining momentum due to their favourable nutritional profile (high in protein and fibre and low in total and saturated fat), environmental benefits, ability to be produced at low cost and resilience to environmental threats, e.g., drought and floods [18]. From a historical stance, fungi have long been used as food source, from truffles to using fungi to ferment drinks, such as beer, sake, and wine [19]. Fungi are a group of eukaryotic organisms that begin as microscopic filaments [20] and are often found living in symbiotic associations with animals, algae, plants and other organisms [21].

In the United Kingdom, interest in mycoprotein first emerged in the 1960s when Lord Rank, a British Industrialist, set out of identify a novel protein source that could be used to attenuate the global food crisis caused by accelerated population growth [22]. Later in 1984, after thorough testing, a derivative of *Fusarium venenatum* (mycoprotein) was authorised for sale by the Ministry of Agriculture, Fisheries and Food in the United Kingdom as a protein food source [23]. Today, mycoprotein is produced at a large-scale vertically using fermentation methods and is exported to at least 15 countries globally, including Australia, Singapore, and the United States under the brand name Quorn^TM^ [24,25,26]. The installation of a fourth fermenter was commissioned in July 2021 which increases capacity to support additional future growth [26]. A full description of mycoprotein production has previously been published by Finnigan (2011) [27].

Alongside well-established production methods, the health evidence for mycoprotein has been accruing. A systematic review of 16 controlled trials concluded that acute mycoprotein ingestion was associated with reductions in total cholesterol, particularly in adults with hyperlipidaemia [28]. An additional systematic review [29] of five randomised controlled trials linked acute mycoprotein intake to lower ad libitum and post-24 h energy intake in healthy lean, overweight, and obese adults. New epidemiological work [30] studying mycoprotein-based food intake using data from the UK National Diet and Nutrition Survey demonstrates that higher mycoprotein intakes were significantly related to lower markers of glycaemia, improved fibre intake profiles and diet quality scores. Taken collectively, an extensive body of evidence has accrued for mycoprotein over the last three decades, emphasising a need to reflect on its integration within FBDG.

## 3. FBDG Alignment and the Incorporation of Protein Sources

FBDG serve as a useful guide to healthy food consumption, thus are central tools for nutrition policy and public health [31]. Now, rising concerns about sustainability have led to the need to appraise FBDG and consider a greater range of aspects within their formulation, which also extends to nutrient supply, links between diet and health, environmental sustainability, energy supply, food-borne contamination and individualisation [31].

In 2019, a global review of FBDG from 90 countries globally concluded that not all messages were universally echoed across all countries [32]. Focusing on protein, countries described what could be termed “protein foods” in a variety of ways [32] demonstrating that inconsistencies were apparent. For example, not all FBDG specifically used the word protein when discussing or displaying what can be termed “protein foods” with 74% including a key message about protein foods, which could include fish (58% of countries), meat (53%), legumes (41%), eggs (31%), poultry (29%), dairy (9%), nuts/seeds (85) and insects (only Kenya) [32]. In total, 11% of countries did not have a protein foods key message that was specific, but instead promoted a generic diversity message, e.g., encouraging consumption from a range of foods as part of a varied diet [32]. It was unclear whether these incorporated mycoprotein, as this was not mentioned specifically, indicating that a specific re-evaluation focusing on fungal proteins and their integration within FBDG could be worthy of future investigation.

Other work similarly concludes that FBDG vary enormously from one country to another [33,34]. A modelling study using data from FBDG from 85 countries demonstrated that most (up to 87% of countries) were incompatible with the Paris Climate Agreement and other environmental targets [34]. These data starkly emphasise the point that FBDG need to become more sustainable, with authors advising that more transparent and specific advice is needed on reducing animal-sourced foods—in the analysis, modulating beef and dairy within FBDG was found to have the greatest effects on improving the environmental sustainability of FBGD [34].

Similarly, a seven-country study (Germany, The Netherlands, USA, India, Oman, Thailand and Uruguay) modelled the carbon footprint of FBDG dietary guidelines from these countries finding that greenhouse gas emissions (GHGE) corresponding to protein food recommendations ranged from 0.03 kg CO_2_-eq/d in India to 1.84 kg CO_2_-eq/d in the United States, for 75 g/d and 156 g/d of protein, respectively [33]. Another review of 43 sets of global FBDG also revealed that environmental impacts of the diet were considered infrequently, particularly in older sets of FBDG which overlooked and aligned less well with environmental and sociocultural aspects of food and diet [35], further emphasising a need for updating. Another European analysis showed that 34 out of 53 countries were found that have formal FBDG with eight “themes” being prominent within these (diet–health relations, nutrient supply, energy supply, dietary habits, sustainability, food-borne contaminants, target group segmentation, and individualization) [31]. Whilst the first four themes appeared to be well-embedded within FBDG, the remaining four, which included sustainability, had almost never been considered [31].

Taken collectively, these findings demonstrate that FBDG are discordant in Europe and globally. This presents a significant challenge and fails to create a level playing field when it comes to minimising the carbon footprint of dietary guidelines. Unless urgent action is taken to harmonise these on a global scale, the ability to use them to assist future public food-related decisions will be seriously misaligned

## 4. Shifts in FBDG

As mentioned, in 2019 the EAT-Lancet Food in the Anthropocene report was central in alerting the need to both nurture human health and reflect on food production systems in relation to their wider environmental impacts [15]. Within this, there are clear messages to limit animal protein and increase plant proteins, but specific guidance regarding fungal proteins are lacking [15]. Some countries have begun to transition and include these aspects within their FBDG. For example, in 2019 France launched new FBDG which considered sustainability and made the recommendation to limit meat except chicken [36]. In the same year, Canada also updated its FBDG and whilst not formally addressing sustainability, it did advise the population to “*choose protein foods that come from plants more often*” defining protein foods as “*legumes, nuts, seeds, tofu, fortified soy beverage, fish, shellfish, eggs, poultry, lean red meat including wild game, lower fat milk, lower fat yogurts, lower fat kefir, and cheeses lower in fat and sodium*” but again with no mention of mycoprotein [37].

In 2021, the Ministry of Environment and Food of Denmark Danish Veterinary and Food Administration (DVFA) also released updated Official Dietary Guidelines [38]. For the first time, these guide Danes on how to eat diets that are healthier, but also more climate friendly [38]. The advice includes to “*eat less meat-choose legumes and fish*” [38]. This is a step in the right direction although the broader concept of “protein diversity” appears to have been overlooked [38]. This provides one example of an organisation moving in the right direction but not yet providing advice that is broad enough to encompass other well-established novel food proteins, such as mycoprotein.

In the Netherlands, the Schijf van Vijf (Wheel of Five in English) has been validated as a tool and food counselling FBDG that can help Dutch adults to adapt their diets to be both healthier and more environmentally sustainable [39,40]. Similarly, this specifies to eat “*less meat and more plant-based food. Vary the diet, switch between fish, pulses, nuts, eggs and vegetarian products*” [39,40]. Whilst this mentions to vary the diet and lists vegetarian products mycoprotein, specifically goes unnoticed [40]. It appears that views about protein are rather dichotomous, e.g., animal versus plant, and other categories such as fungal-derived mycelium are comparatively overlooked [4].

Other research further focusing on Dutch eating habits showed that a distinct shift away from beef, butter, pork and cheese, and movements towards the consumption of legumes, peanuts and tree nuts, fish, shellfish, and soy foods, would be needed to achieve GHG emission targets [2]. However, complying and achieving the lowest GHG emissions target resulted in a diet that lacked food diversity and was susceptible to nutritional inadequacies [2]. Subsequently, authors concluded that innovations in food production and processing would be needed [2]. Again, this further emphasises the need to embed the concept of “protein diversity” within updated sustainable sets of FBDG.

In the UK, it has been reported that complying with the UK Eatwell Guide would help to reduce GHG emissions of current adult diets by 30%, and reduce water use by 4% [41]. These conclusions were drawn from a review of 29 studies that adopted a range of methodologies to define healthier, sustainable diets [42]. In this publication, it was concluded that protein diversity is one means of making diets healthier and more sustainable with the inclusion of beans, pulses, nuts, seeds, soya and mycoprotein being some actions that people could take [41,42]. This is a positive move forward, although these shifts remain to be formally embedded within the Eatwell Guide infographic.

The Nordics are also in the process of updating their Nordic Nutrition Recommendations for 2022, and are calling out sustainability with a vision of making the Nordic region the most sustainable and integrated region in the world [43].

## 5. Protein Quality Concept

Proteins are a macronutrient and central part of the human diet, chemically consisting of carbon, hydrogen, nitrogen, and oxygen [44]. Dietary proteins are found in animal foods, plant foods and single-cell organisms (e.g., those of algal, bacterial, mould, or yeast origin) and are present in different proportions with variable amino acid profiles [45]. In the past, the concept of protein quality has focused solely on “a protein’s ability to provide specific patterns of amino acids to satisfy the demands for synthesis of protein as measured by animal growth or, in humans, nitrogen balance” [46]. Due to the fact that protein quality metrics are based on the profiles of essential amino acids and their digestibility, these metrics have then been taken up and embedded by some national and international regulatory agencies as a means of developing protein content and quality claims for food marketing purposes [47]. Yet, in modern times the term “quality” lacks resolution, which can lead to misleading interpretations [48]. The Protein Digestibility-Corrected Amino Acid Score (PDCAAS) was adopted by the Food and Agriculture Organization (FAO)/World Health Organization (WHO) and is the preferred method for determining protein quality from foods [49]. The PDCAAS is a method of evaluating protein quality, typically expressed as a percentage (mg of limiting amino acid in 1 g of test protein/mg of same amino acid in 1 g of reference protein) × faecal true digestibility percentage) [49]. Yet, when adopting such methods, animal proteins (>95% digestibility) tend to come out more favourably than certain plant proteins (50–80% digestibility) due to higher digestibility and a distribution of the nine essential amino acids regarded as being better aligned with human requirements [45,50].

More recently, the digestible indispensable amino acid score (DIAAS) has been developed as an alternative approach to measuring protein quality [51]. This approach is supported by the Food and Agriculture Organization of the United Nations and challenges concerns over the practise of true faecal digestibility as a marker of amino-acid digestibility in the PDCAAS methodology, and instead uses ileal amino acid digestibility coefficients and untruncated protein scores [51]. Whilst the DIAAS is now increasingly being advocated as an updated method for determining protein quality by FAO Experts [52,53], just like the PDCAAS this still fails to consider broader issues of relevance, such as longer-term measures of health and environmental ramifications. It also suffers from the limitation that it is not necessarily applicable in humans, as it cannot properly be measured in humans

## 6. Shifting Trends, Shifting Concepts

Both PDCAAS and DIAAS have not yet “hit the mark” and encompassed the “net health effect” (which includes environmental effects) of protein foods [48]. The valorisation of traditional animal-based protein sources means that the public and consumer-facing messages now need to encompass health and environmental outcomes when we refer to protein quality. Katz et al. [48] proposed a new definition of protein quality compiled into a metric that could be applied to national and food regulatory labelling systems. This focuses on three dimensions: (1) the concentration of protein and individual amino acids in the food, e.g., derived using PDCAAS), (2) an assessment of the evidence of health outcomes associated with consumption of the food, e.g., nitrogen balance or lipid profiles and (3) an assessment of potential environmental impacts of producing the food. This rounded approach could help scientists and consumers alike to better recognise the value of alternative proteins, e.g., plant and fungal derived, and could allow people to eat for their own health and the health of the environment. However, no movements have yet been made to take this on board.

Mycoprotein is regarded as a “complete protein” due to it providing all nine essential amino acids and having a protein digestibility-corrected amino acid score of 0.996 (derived using gold-standard ileostomy methods) [54]. Previously, a series of five trials concluded that mycoprotein was a bioavailable protein that could stimulate muscle protein synthesis rates [55]. In these trials, mycoprotein appeared to represent a bioavailable and insulinotropic protein food source, resulting in slower and more sustained hyperinsulinaemia and hyperaminoacidaemia compared with protein-matched milk [55]. Other work has further shown that 70 g of mycoprotein (31.5 g protein: 2.5 g leucine) stimulated both resting and postexercise muscle protein synthesis rates in resistance-trained young males, and this was to a level greater than a leucine-matched bolus of milk protein [56]. Most recently, a 3-day intervention has shown that vegan-derived dietary protein (primarily in the form of mycoprotein) compares with animal-derived protein and can support both rested and exercised daily myofibrillar protein synthesis rates (MPS) in healthy aged adults (mean age 66 years) consuming a high-protein diet [57]. In particular, daily MPS rates were 13 and 12% greater in the exercised versus rested leg in the omnivorous diet and the vegan diets, respectively, indicating that the different diets had similar effects on MPS [57].

Mycoprotein is considered to be “high in protein” as per the requirements set out in the European Commission’s nutrition claims legislation, i.e., at least 20 per cent of the energy value of the food is provided by protein [58,59]. As shown in Table 1 mycoprotein provides all nine essential aminos acids. Levels of total essential amino acids are 21.1 g/100 g, thus higher than lean cooked beef and skinless cooked chicken (14.8 and 14.1, respectively). Levels of branched-chain amino acids (9.1 g/100 g) are also higher than other foods typically classed as being “protein foods”, which ranged from 1.1 g/100 g for chickpeas to 6.2 g/100 g for lean, cooked beef. These data reinforce underpinning research and demonstrates that mycoprotein is a well-established and valuable food protein source that should be incorporated within FBDGs.

## 7. Discussion

In the 21st century, adequate protein intakes remain essential for longevity, metabolic and muscular health [61,62]. Now, given COP26 and the urgency to achieve the 2016 Paris Agreement “to commit and reduce greenhouse gas emissions by at least 40% by 2030”, there has been a vast upsurge in recognition that plant proteins are preferable from a planetary resource perspective and more sustainable than animal-derived proteins [15,63,64]. Plant proteins, however, are just one way forward. Other environmentally friendly meat alternatives such as mycoprotein which are high in protein, provide essential amino acids, high in fibre, low in total and saturated fat and deliver a range of micronutrients, also need to be considered [22,65]. Failure to recognise this appears to be an unconscious omission.

Perceptions of “protein quality” are now discordant with these shifts. As discussed, protein quality definitions are becoming inadequate as society begins to transition towards diets containing a diverse range of food proteins (i.e., moving beyond animal-derived food proteins) [48]. Recent evidence now shows that meat alternatives—plant-based and fungal-derived mycoprotein—can support the maintenance of muscle mass/myofibrillar protein synthesis rates in populations of older adults [57,66]. In 2019, Katz and coauthors proposed that protein quality definitions were antiquated, providing an example of an updated model that encompassed both health and environmental outcomes [48]. Yet, approaching three years on, no movements have been undertaken to review the proposal. It is therefore recommended that basic protein quality definitions are re-evaluated and built upon, providing more than a sole assessment of protein digestibility. Certainly, if health and environmental outcomes were factored in, the concept of protein quality would begin to align more constructively with environmental movements.

The same, to an extent, also applies to FBDG. We have seen globally how these are interchangeable in terms of which protein sources are included and recommended for consumption. Even just within Europe, it has been confirmed that the level of detail and quality of FBDG vary considerably in relation to specification of frequency and portion size of food proteins and recommendations for specific target groups and time of last update [67]. Amongst those that have been updated, these have a tendency to include “plant-based” food proteins but overlook other nonanimal sources of proteins [32]. For mycoprotein, more than 16 controlled trials have studied inter-relationships with health, with evidence indicating that this is a bioavailable source of amino acids with the ability to promote muscle protein synthesis and reinforce human health [28]. Mycoprotein subsequently has a large body of clinical trials which justify its inclusion within future sustainable sets of FBDG.

Given this, now is an appropriate time to consider the concept of “protein variety”. Globally, current food systems lack diversity and are reliant on just a small range of foods, with approximately 75% of global food supplies being derived from around five animal and 12 plant species [68,69], with little mention of fungal species. Novel precision fermentation yielding mycoprotein is a viable third category of protein that could be better utilised to meet growing protein demands [68].

Consequentially, we should be less black and white in our approach. There is present generic tendency to focus on eating animal- or plant-derived protein. Instead, we should think about moving towards the concept of “protein diversification”. Livestock-derived protein, aquaculture-derived protein, plant protein, marine protein, e.g., algae/seaweed, insects, protein from fermentation, e.g., mycoprotein and meat analogues [68,70] can all provide protein. This approach would embody a greater range of food options for the consumer, whilst the ratios in which these are consumed can be adjusted to align with health and sustainability policies. Certainly, alongside animal- and plant-derived food proteins, fungal proteins are now a well-established and viable protein food source that could be added to the list [71].

Taken together, updating both protein quality definitions and FBDG would help to better inform consumers. In turn, this would go some way towards helping transition consumers acceptance of alternative proteins. At present, outdated definitions and inconsistent FBDG can only be confusing to public sectors. A recent systematic review of 91 studies concluded that attitudes, familiarity, taste and health, food neophobia and social norms were some of the main drivers affecting acceptability of alternative proteins [72]. Cognitive claims also appeared to be of interest to the consumer [72]. In summary, several changes are proposed:

- Redundant definitions of protein quality (PDCAAS and DIAAS) should be re-evaluated and potentially “layered” to factor in health and environmental outcomes in addition to protein digestibility-corrected amino acid scores per se.
- FBDG now need to move away from the “black and white” approach—animal-derived versus plant-derived protein—and instead focuses on the concept of “protein diversity", i.e., that diets should contain less animal-derived food protein and instead aim to include a variety of other foods providing protein, e.g., plant-based, fungal (mycoprotein) and others, e.g., algal, or insect-derived food proteins. To progress to such a model, multidisciplinary data from additional protein sources will need to be evaluated.
- There needs to be a greater consensus between FBDG across the globe. Misalignment of FBDG and improvements to these in some countries is not going to have a hard-hitting effect on overall food-derived carbon emissions. Thus, global food policies are warranted and would facilitate adherence at the population level. We subsequently call for protein derived from filamentous fungi to be incorporated within the EAT Lancet 2.0 and forthcoming Nordic Dietary Guidelines.
- It is believed that changes to the above would result in clearer, more consistent public health messaging globally, and in turn, facilitate successful change in transitioning to sustainable diets at the consumer level.

## 8. Conclusions

In concluding this article, the time of change is upon us. Whilst we once may have discounted where our food proteins came from, environmental drivers are changing this with rapidity. Prior protein quality definitions are now antiquated, and views about protein need to shift and encompass both health and environmental outcomes. It is proposed that both protein quality definitions and FBDG should be updated. These need to focus on health, environmental outcomes, and integrate the concept of protein diversity. This would then enable other well-established food proteins such as mycoprotein to be included.

## Figures and Tables

**Table 1:** Amino Acid Profile of Mycoprotein and other Protein Foods.

Amino Acids	g Amino Acids per 100 g								
	Mycoprotein (dw) [11,55]	Beef, Lean Only, Cooked, Braised [60]	Chicken Breast, Skinless, Meat Only, Cooked [60]	Egg, Whole, Cooked, Hard-Boiled [60]	Chickpeas, Canned, Drained Solids [60]	Kidney Beans, Drained Solids [60]	Lentils, Cooked, Boiled, without Salt [60]	Almonds, Nuts [60]	Pistachio Nuts [60]
Alanine	2.8	2.2	1.9	0.7	0.3	0.4	0.4	1.0	1.0
Arginine	3.3	2.3	2.2	0.8	0.7	0.4	0.7	2.5	2.1
Aspartic acid	4.6	3.3	3.0	1.3	0.8	1.0	1.0	2.6	1.9
Cystine	0.4	0.4	0.3	0.3	0.1	0.1	0.1	0.2	0.3
Glutamic acid	5.6	5.4	4.8	1.6	1.2	1.3	1.4	6.2	4.3
Glycine	2.0	2.0	1.4	0.4	0.3	0.3	0.4	1.4	1.0
Histidine *	1.6	1.2	1.2	0.3	0.2	0.2	0.3	0.5	0.5
Isoleucine **	2.4	1.6	1.6	0.7	0.3	0.4	0.4	0.8	0.9
Leucine **	3.9	2.9	2.7	1.1	0.5	0.7	0.7	1.5	1.6
Lysine *	3.8	3.0	3.1	0.9	0.5	0.6	0.6	0.6	1.1
Methionine *	1.0	0.9	0.8	0.4	0.1	0.1	0.1	0.2	0.4
Phenylalanine *	2.3	1.4	1.3	0.7	0.4	0.5	0.4	1.1	1.1
Proline	2.0	1.6	1.0	0.5	0.3	0.5	0.4	1.0	0.9
Serine	2.3	1.4	1.2	0.9	0.4	0.5	0.4	0.9	1.3
Threonine *	2.5	1.6	1.4	0.6	0.3	0.3	0.3	0.6	0.7
Tryptophan *	0.8	0.4	0.4	0.2	0.1	0.1	0.1	0.2	0.3
Tyrosine	1.8	1.2	1.2	0.5	0.2	0.2	0.2	0.5	0.5
Valine **	2.8	1.8	1.7	0.8	0.3	0.5	0.4	0.9	1.3
EAAs	21.1	14.8	14.1	5.6	2.6	3.3	3.3	6.3	7.8
NEAAs	24.8	19.8	17.0	7.0	4.3	4.6	5.0	16.3	13.3
BCAAs	9.1	6.2	5.9	2.5	1.1	1.5	1.5	3.1	3.8
Data source/food ID	Dunlop et al. (2017) [55]; Coelho et al. (2020) [11]	170,235	171,140	173,424	173,800	174,285	172,421	170,567	170,184

Key: BCAAs, branched-chain amino acids; dw, dry weight; * EAAs; b, branched-chain amino acids.; ID, identification; ** NEAAs, nonessential amino acids.
